# Targeting loss of the Hippo signaling pathway in *NF2*-deficient papillary kidney cancers

**DOI:** 10.18632/oncotarget.24112

**Published:** 2018-01-10

**Authors:** Carole Sourbier, Pei-Jyun Liao, Christopher J. Ricketts, Darmood Wei, Youfeng Yang, Sarah M. Baranes, Benjamin K. Gibbs, Lernik Ohanjanian, L. Spencer Krane, Bradley T. Scroggins, J. Keith Killian, Ming-Hui Wei, Toshiki Kijima, Paul S. Meltzer, Deborah E. Citrin, Len Neckers, Cathy D. Vocke, W. Marston Linehan

**Affiliations:** ^1^ Urologic Oncology Branch, CCR, National Cancer Institute, NIH, Bethesda, MD, USA; ^2^ Radiation Oncology Branch, CCR, National Cancer Institute, NIH, Bethesda, MD, USA; ^3^ Genetics Branch, CCR, National Cancer Institute, NIH, Bethesda, MD, USA

**Keywords:** NF2, PRCC, hippo pathway, yes, dasatinib

## Abstract

Papillary renal cell carcinomas (PRCC) are a histologically and genetically heterogeneous group of tumors that represent 15–20% of all kidney neoplasms and may require diverse therapeutic approaches. Alteration of the *NF2* tumor suppressor gene, encoding a key regulator of the Hippo signaling pathway, is observed in 22.5% of PRCC. The Hippo signaling pathway controls cell proliferation by regulating the transcriptional activity of Yes-Associated Protein, YAP1. Loss of *NF2* results in aberrant YAP1 activation. The Src family kinase member Yes also regulates YAP1 transcriptional activity. This study investigated the importance of YAP and Yes activity in three *NF2*-deficient PRCC cell lines. *NF2*-deficency correlated with increased expression of YAP1 transcriptional targets and siRNA-based knockdown of YAP1 and Yes1 downregulated this pathway and dramatically reduced cell viability. Dasatinib and saracatinib have potent inhibitory effects on Yes and treatment with either resulted in downregulation of YAP1 transcription targets, reduced cell viability, and G0-G1 cell cycle arrest. Xenograft models for *NF2*-deficient PRCC also demonstrated reduced tumor growth in response to dasatinib. Thus, inhibiting Yes and the subsequent transcriptional activity of YAP1 had a substantial anti-tumor cell effect both *in vitro* and *in vivo* and may provide a viable therapeutic approach for patients with *NF2*-deficient PRCC.

## INTRODUCTION

Papillary renal cell carcinoma (PRCC) is the second most common type of kidney cancer representing approximately 15–20% of all cases. PRCC is heterogeneous and categorized into two main histologic subtypes, type 1 PRCC and type 2 PRCC [1, 2]. PRCC can present as both sporadic disease or as a component of a hereditary syndrome, such as hereditary papillary renal cell carcinoma (HPRC) or hereditary leiomyomatosis and renal cell carcinoma (HLRCC) [3]. HPRC patients carry germline activating mutations within the tyrosine kinase domain of the *MET* oncogene and presents with bilateral, multifocal type 1 PRCC [4, 5], while HLRCC patients inherit germline inactivating mutation of the *FH* tumor suppressor gene and can have an aggressive variant of type 2 PRCC [6, 7]. Although numerous studies have been done on familial forms of PRCC, little was known about the genes responsible for the sporadic forms of PRCC until a recent report from the Cancer Genome Atlas (TCGA) in which 161 PRCC tumors were sequenced and analyzed [2]. This study highlighted that amplification of chromosome 7 and 17 and activating mutations of the MET gene, on chromosome 7, are associated with type 1 PRCC, while type 2 PRCC was more genetically diverse including tumors that had either germline or somatic *FH* mutation, chromosomal translocations producing *TFE3*-fusions, or NRF2 pathway mutations in *NFE2L2* (NRF2) or *CUL3* [2]. In addition, whole genome sequencing of all PRCC types identified five frequently mutated genes, *MET*, *SETD2*, *NF2*, *KDM6A*, and *SMARCB1*, suggesting that alterations to the MET signaling pathway, Hippo signaling pathway, and the chromatin modifier pathways may play an important role in PRCC carcinogenesis [2]. Currently, there are no standard of care therapies for most patients with advanced PRCC and clinical trials have been limited to targeting the MET signaling pathway [8].

Neurofibromatosis type 2 (*NF2*) is a tumor suppressor gene that regulates the Hippo signaling pathway, which normally controls organ growth by suppressing cell division and proliferation and activating programmed cell death/apoptosis [9]. Heterozygous germline inactivating mutations of *NF2* are responsible for the hereditary tumor predisposition syndrome neurofibromatosis type 2, which is characterized by the development of multiple tumors in the nervous system [10, 11]. Somatic mutation or loss of the *NF2* gene have been previously reported at a low frequency in a breast cancer, prostate cancer, clear cell renal cell carcinoma, and PRCC [2, 12, 13]. The Hippo signaling pathway is evolutionary conserved and regulates tissue growth and survival by transmitting extracellular signals such as contact inhibition to the cells. Cell-to-cell contact activates the Hippo signaling cascade through proteins such as Merlin (encoded by *NF2*) that induces the MST1/2 complex, with the regulatory protein Salvador, to phosphorylate the LATS1/2/MOB1 complex that leads to the phosphorylation dependent inhibition of the transcriptional activity of Yes-associated protein 1 (YAP1) and transcriptional co-activator with PDZ-binding motif (TAZ) [9]. Thus, the *NF2* gene product Merlin suppresses the transcriptional activity of YAP1 and controls contact inhibition of normal cells. Complete loss of the *NF2* gene would lead to an inability to suppress the activity of YAP/TAZ, driving the expression of multiple downstream transcription targets, including *BIRC5* (survivin), *CTGF* (connective tissue growth factor), and *CCND1* (cyclin D1), that all promote cell growth and survival [9]. Aberrant YAP1 transcriptional activity can also be driven in β-catenin active tumors via the activation of the Src family tyrosine-protein kinase Yes. Yes activates the YAP1-β-catenin-TBX5 complex transcriptional activity and promotes the survival of the cells by increasing expression of multiple genes, such as *BIRC5* [14]. Yes inhibitors, such as dasatinib and saracatinib [15], have been shown to be active in preclinical studies of cancer cell lines with increased YAP1 transcription target expression due to increased β-catenin [14, 16] and therefore, could be an attractive potential therapeutic target in cancer cell lines with a deficiency in the Hippo signaling pathway.

In this report, we investigated the effect on the growth and survival of *NF2*-deficient PRCC cell lines of targeting YAP1 transcription activation resulting from the Hippo signaling pathway loss. We investigated three PRCC cell lines bearing *NF2* mutations and assessed the effect of Yes inhibitors on their proliferation and survival *in vitro* and *in vivo* with the goal of developing a novel potential therapeutic approach for PRCC patients whose tumors are characterized by an *NF2* deficiency.

## RESULTS

### The Hippo signaling pathway is altered in 22.5% of PRCC

**Figure 1:**
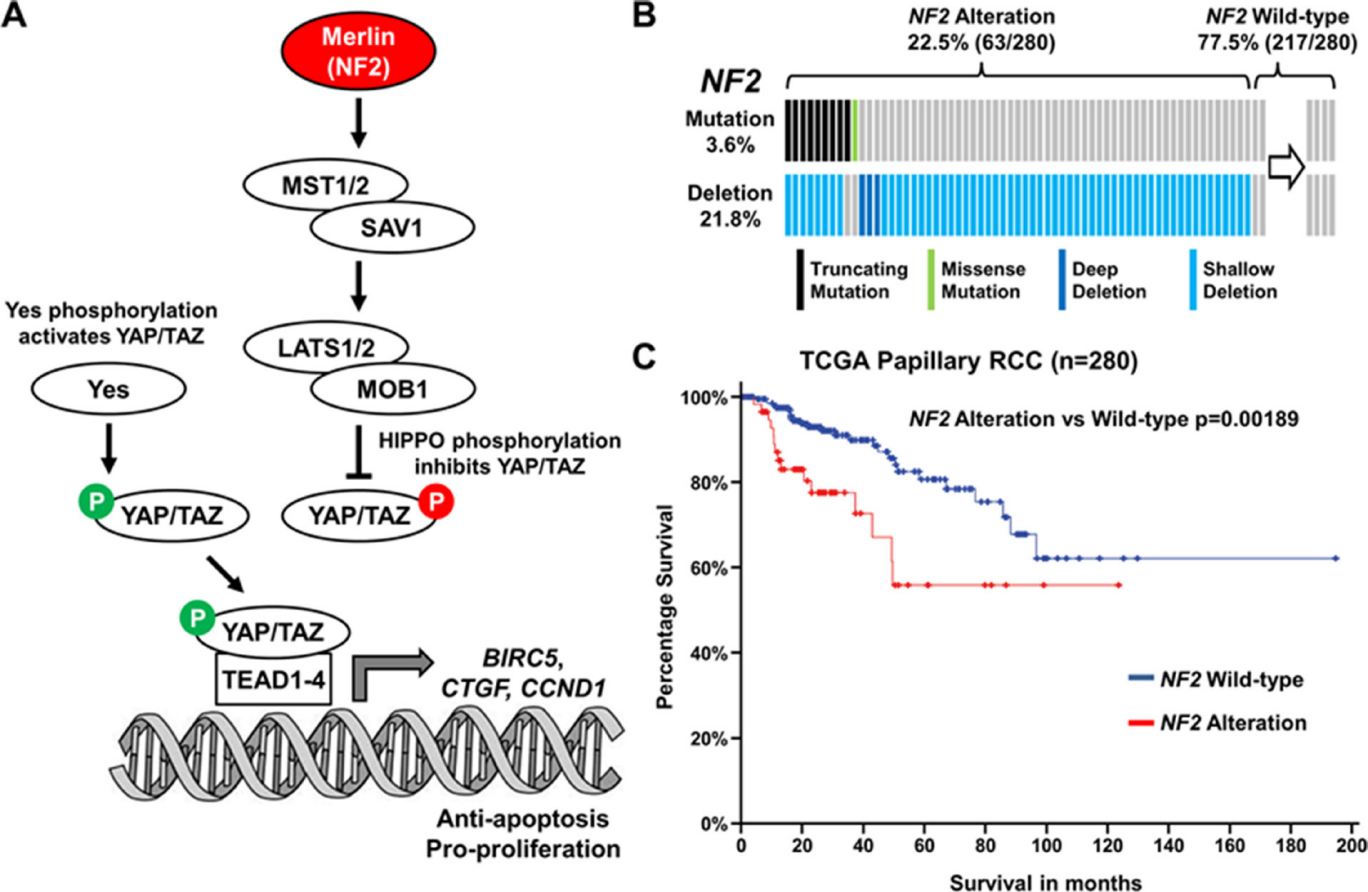
Hippo signaling pathway gene *NF2* is altered in 22.5% of PRCC (**A**) Cartoon depicting the Hippo signaling pathway and how Merlin (encoded by *NF2*) and tyrosine-protein kinase Yes play a role in this pathway. (**B**) Mutation and deletion of *NF2* in the TCGA cohort of 280 PRCC tumors (Data downloaded for cBioPortal [17, 18]). (**C**) Survival of patients with *NF2* altered PRCC tumors compared to *NF2* unaltered PRCC tumors.

The Cancer Genome Atlas (TCGA) data has recently shown that *NF2* is among the five most commonly mutated genes in PRCC and was present in 3.6% of cases (10/280–cBioPortal; Figure 1A–1B) [17, 18]. Furthermore, copy number loss of the *NF2* gene was observed in 21.8% of cases with most mutation positive cases also demonstrating copy number loss (Figure 1B). While the presence of a *NF2* mutation did not correlate with survival, the 22.5% of PRCC cases that demonstrated either *NF2* mutation or copy number loss demonstrated a strong correlation with poorer survival (*p* = 0.00189; Figure 1C). These data suggest that dysregulation of the Hippo signaling pathway might be a critical driver for PRCC growth.

**Figure 2:**
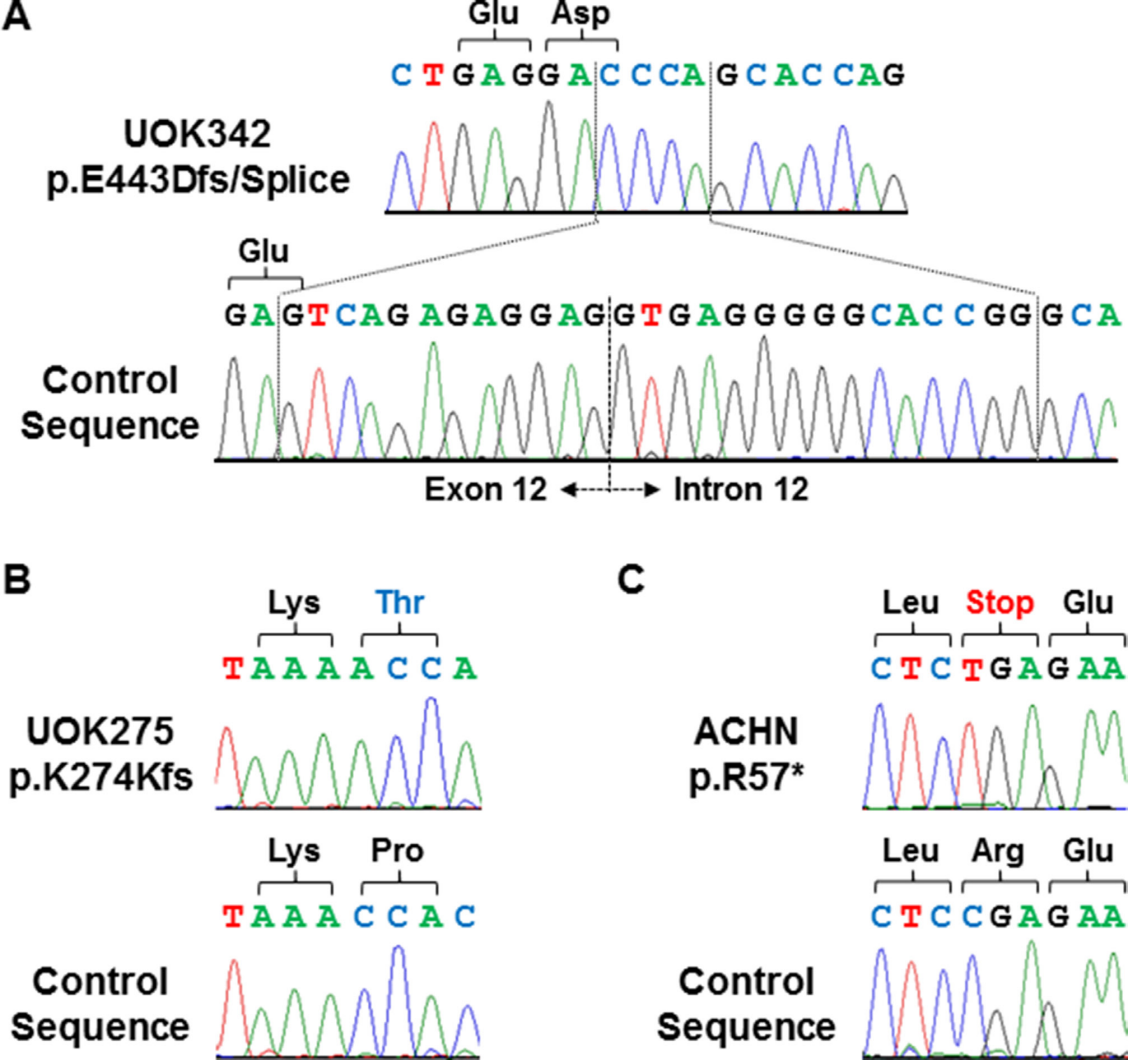
Homozygous mutations of *NF2* in 3 PRCC cell lines Homozygous frameshift and truncation mutations of *NF2* were demonstrated by Sanger sequencing in UOK342 (**A**), UOK275 (**B**), and ACHN (**C**). DNA extracted from normal blood samples were used to generate control sequences.

To provide a cell line model of Hippo signaling pathway loss, the patient-derived cell lines generated at the Urologic Oncology Branch from PRCC patient material were screened for complete mutational loss of the *NF2* gene. This identified one cell line derived from a patient with type 1 PRCC, UOK342, with a homozygous *NF2* insertion/deletion alteration that resulted in frameshift mutation and the loss of a splice site (Figure 2A) and one cell line derived from a patient with type 2 PRCC, UOK275, with a homozygous *NF2* single nucleotide insertion that resulted in frameshift mutation (Figure 2B). These two newly identified *NF2*-deficient cell lines were combined with the existing ACHN cell line, known to have a homozygous *NF2* stop codon mutation (https://cansar.icr.ac.uk/cansar/cell-lines/ACHN/mutations/) (Figure 2C), for further study.

### Hippo signaling pathway loss in *NF2*-deficient PRCC cells supports their survival through YAP1 activation

**Figure 3:**
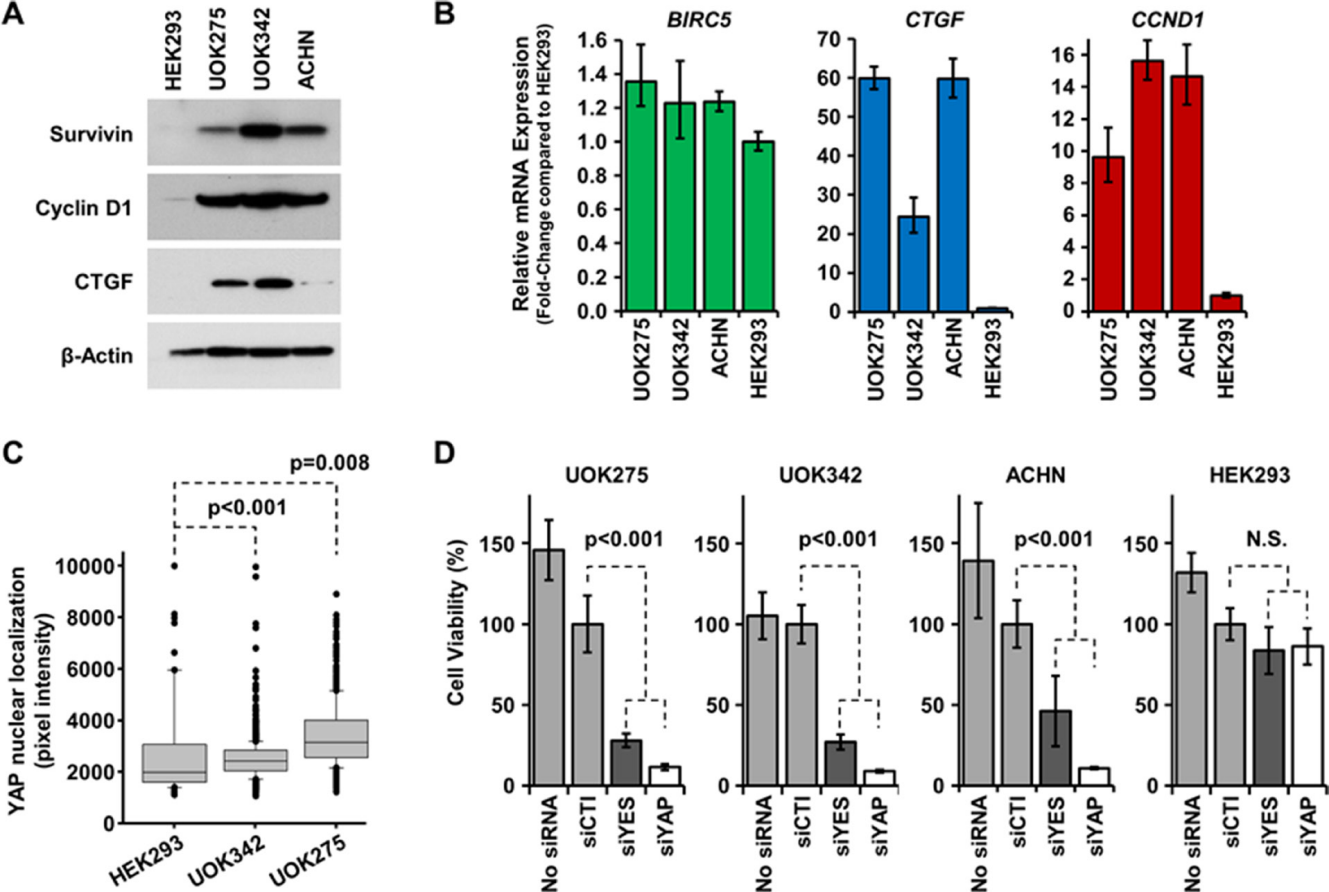
Inhibition of the Hippo pathway is cytotoxic to *NF2*-deficient tumor cells (**A**) Basal protein expression level of YAP1 transcription targets survivin, cyclin D1 and CTGF were assessed by immunoblotting in UOK275, UOK342 and ACHN with HEK293 used to represent normal levels of expression. Β-actin expression levels were used as a loading control. (**B**) Gene expression of *BIRC5* (survivin), *CTGF* (CTGF) and *CCND1* (cyclin D1) in UOK275, UOK342 and ACHN. HEK293 were used as controls. (**C**) YAP1 nuclear localization in HEK293, UOK275, and UOK342 was quantified using an InCell Analyzer. (**D**) The effect of transient silencing of *YES1* and *YAP1* by small interference RNA or mock siRNA on cell viability was assessed in UOK275, UOK342, ACHN and HEK293 72 hours post-transfection by Cell-Titer Glo assay. N.S.: non-significant; siCTL: mock siRNA; siYES: siRNA against *YES1*; siYAP: siRNA against *YAP1*.

*NF2* alteration leads to the activation of the transcription factor YAP1 and the expression of multiple downstream targets promoting tumor cell survival and proliferation [9, 19]. Since homozygous mutation of *NF2* was found in UOK275, UOK342 and ACHN, we investigated whether these alterations led to the expression of YAP1 downstream targets. As shown on Figure 3A–3B, survivin, cyclin D1 and CTGF were all overexpressed at the protein and mRNA levels compared to HEK293. Since all three genes can be transcribed by multiple transcription factors, we next measured YAP1 nuclear localization in *NF2*-deficient PRCC cells and HEK293 cells using a high content imaging system (InCell 2000 Analyzer). As shown in Figure 3C, YAP1 had a significantly higher nuclear localization in *NF2*-deficient PRCC cell lines compared to HEK293 cells suggesting that YAP1 is more activated in *NF2*-deficient PRCC cell lines (representative images are shown in Supplementary Figure 1A). Taken together, these data demonstrate that the Hippo pathway is activated in UOK275, UOK342 and ACHN.

Tyrosine-protein kinase Yes, encoded by the *YES1* gene, is one of the main regulators of YAP1, in addition to the Hippo signaling pathway, and drives transcriptional activity in β-catenin active tumors [14]. To assess whether loss of the Hippo signaling pathway due to *NF2* mutation may provide a therapeutic target, either YAP1 or its regulator Yes were transiently silenced using small interference RNAs (Supplementary Figure 1B). Transient silencing of either *YAP1* or *YES1* proved to be cytotoxic to UOK275, UOK342 and ACHN cells, while there was no effect on the survival of HEK293 (Figure 3D). This suggests that inhibition of the Hippo pathway in *NF2*-deficient PRCC may have a therapeutic value and may selectively target the tumor cells.

### Inhibition of Yes-mediated YAP1 activation is cytotoxic to *NF2*-deficient PRCC cells

**Figure 4:**
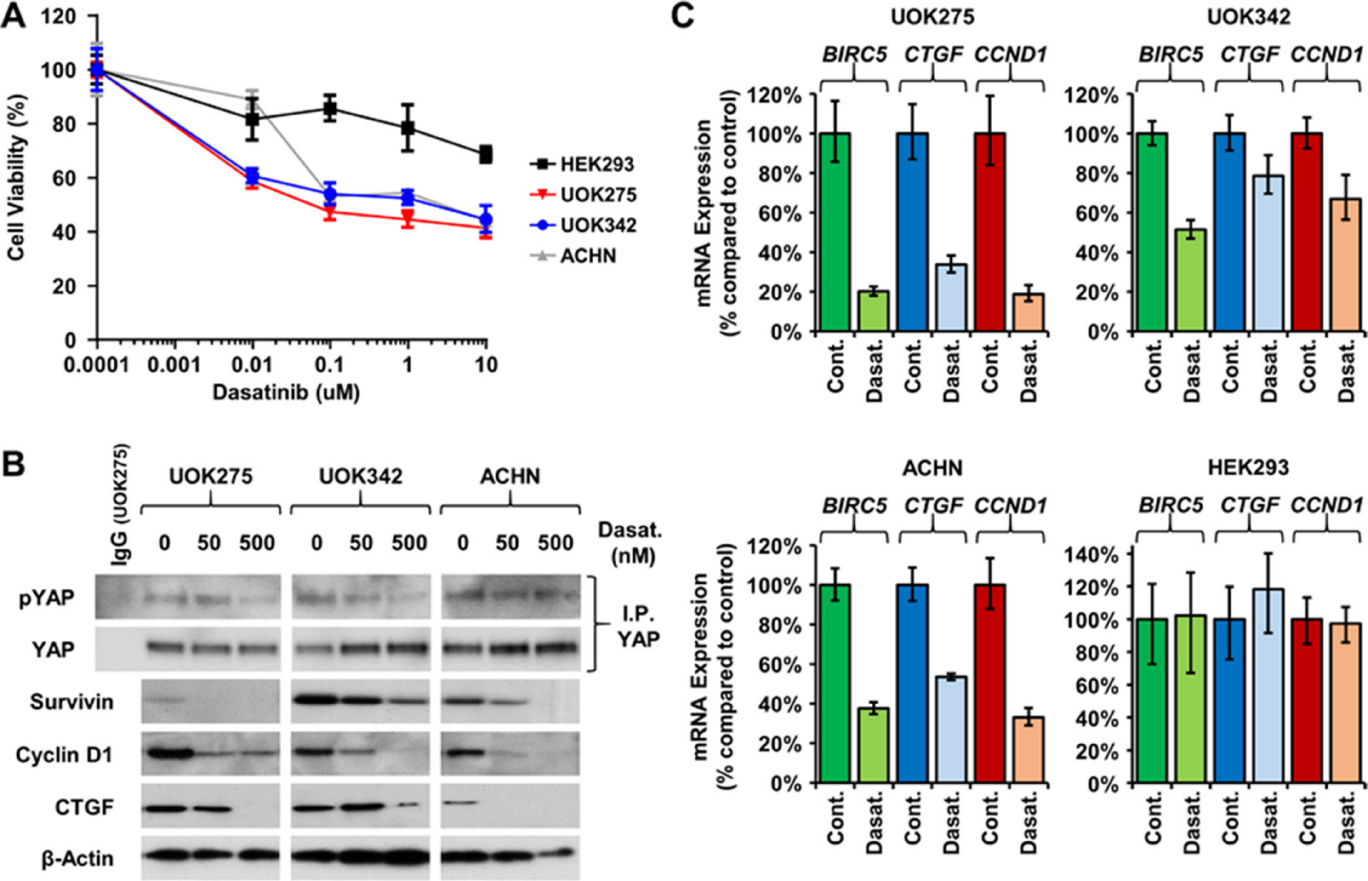
Yes inhibitors lower YAP1-mediated transcriptional network (**A**) Dose concentration treatment of dasatinib (μM) on the viability of UOK275, UOK342 and ACHN. HEK293 were used as controls. Cells were treated as indicated for 48 hours then viability was assessed by Cell-Titer Glo assay. (**B**) Yes-dependent phosphorylation of YAP1 and protein expression of survivin, cyclin D1 and CTGF was evaluated following 24 hours of dasatinib treatment by immunoblotting. (**C**) Expression of *BIRC5* (survivin), *CTGF* (CTGF) and *CCND1* (cyclin D1) was assessed by TaqMan assay 24 hours after dasatinib treatment (100nM) in HEK293, UOK275, UOK342 and ACHN.

Yes is one of nine members of the Src kinase family along with Src, Fyn, Lyn, ABL, and Lck. Despite the role of Yes in regulating the Hippo signaling pathway through phosphorylation of YAP and supporting tumor growth, there are currently only a small proportion of the clinically approved Src kinase family inhibitors that effectively target Yes, such as dasatinib and saracatinib. Dasatinib is a Yes, Bcr-ABL, Src, and Kit inhibitor, and has been approved by the Food and Drug Administration for the treatment of chronic phase Philadelphia chromosome-positive chronic myelogenous leukemia (CP-CML) and Philadelphia chromosome-positive acute lymphoblastic leukemia (ALL) [20–22]. Saracatinib is a known inhibitor of Fyn, Yes, Src, Bcr-ABL and Lck, and has recently been repurposed for the treatment of Alzheimer's disease [23]. Since silencing of *YES1* was lethal to *NF2*-deficient PRCC tumor cells, their survival was assessed following dasatinib and saracatinib treatment. Both decreased the survival of *NF2*-deficient PRCC (Figure 4A and Supplementary Figure 2A) as well as the expression of YAP1 downstream targets (Figure 4B–4C, Supplementary Figure 2B). The embryonic kidney cell line HEK293 used as control was unaffected by the treatments (Figure 4A and 4C). To distinguish the specific effects of Yes inhibition from addition effects due to inhibition of other Src kinase family members, two alternative Src kinase family inhibitors, PP2 and WH-4-023, that do not inhibit Yes were used. They did not affect cell viability or alter the expression of the YAP1 downstream target genes (Supplementary Figure 2A–2B). The levels of Yes-dependent phosphorylation on YAP1 (Tyr357) were also assessed by immunoblotting. As shown in Figure 4B, dasatinib decreased Yes-dependent phosphorylation of YAP1 in all 3 *NF2*-deficient cell lines.

### Inhibition of Yes arrests the cell cycle of *NF2*-deficient PRCC cells

**Figure 5:**
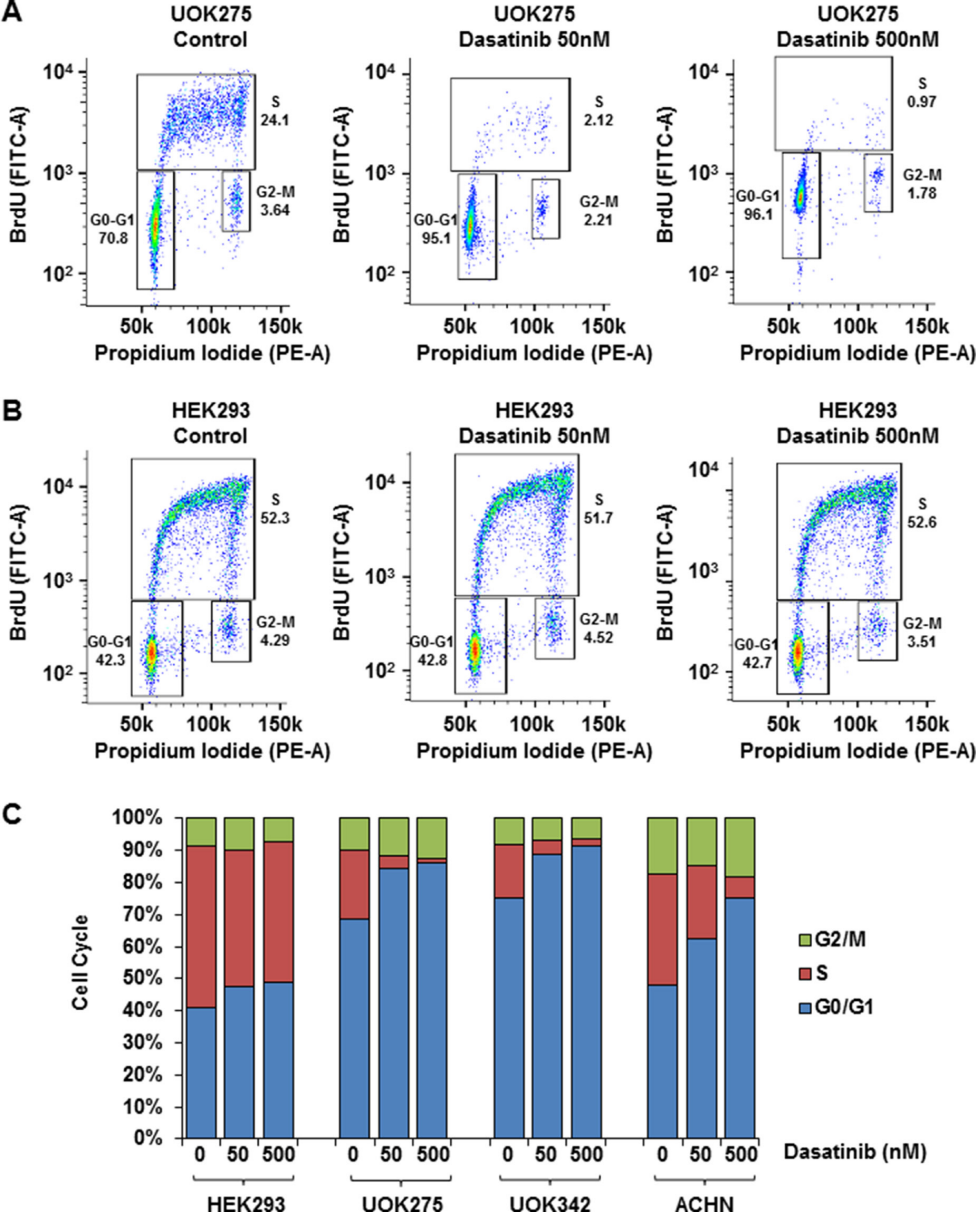
Yes inhibitors block *NF2*-deficient PRCC cell cycle Quantification of UOK275 (**A**) and HEK293 (**B**) cell cycle phases by BrdU and propidium iodide (PI) staining. The panels display the position of gates used to quantify cells in G0-G1, S or G2-M phases after 18 hours of treatment with DMSO (control), or dasatinib (50 nM or 500 nM). (**C**) Quantification of cells in the different cell cycle phases in HEK293, UOK275, UOK342 and ACHN after DMSO or dasatinib treatment (average of 3 distinct experiments).

In normal cells, the balance between cell cycle arrest and cell proliferation is tightly regulated by extracellular signals and contact inhibition. The Hippo signaling pathway is critical in mediating those signals, thus alteration of *NF2* and YAP1 activation lead to aberrant cell proliferation and loss of contact inhibition [24]. To investigate how dasatinib and saracatinib affect cell cycle, *NF2*-deficient cells were treated with either 50 nM or 500 nM of dasatinib or 100 nM of saracatinib, stained with BrdU and propidium iodide and analyzed by flow cytometry. As shown in Figure 5A–5C, inhibition of YAP1 with dasatinib led to a G0/G1 arrest in *NF2*-deficient cell lines but did not affect the cell cycle of HEK293 (see also Supplementary Table 1). Similar results, although less pronounced, were observed with saracatinib (Supplementary Table 2). Src inhibitors PP2 and WH-4-023 did not affect the cell cycle of *NF2*-deficient cells suggesting that the previous observed effect is likely to be Yes-mediated (Supplementary Tables 3 and 4 respectively).

### Dasatinib inhibits tumor growth in two *NF2*-deficient xenograft animal models

**Figure 6:**
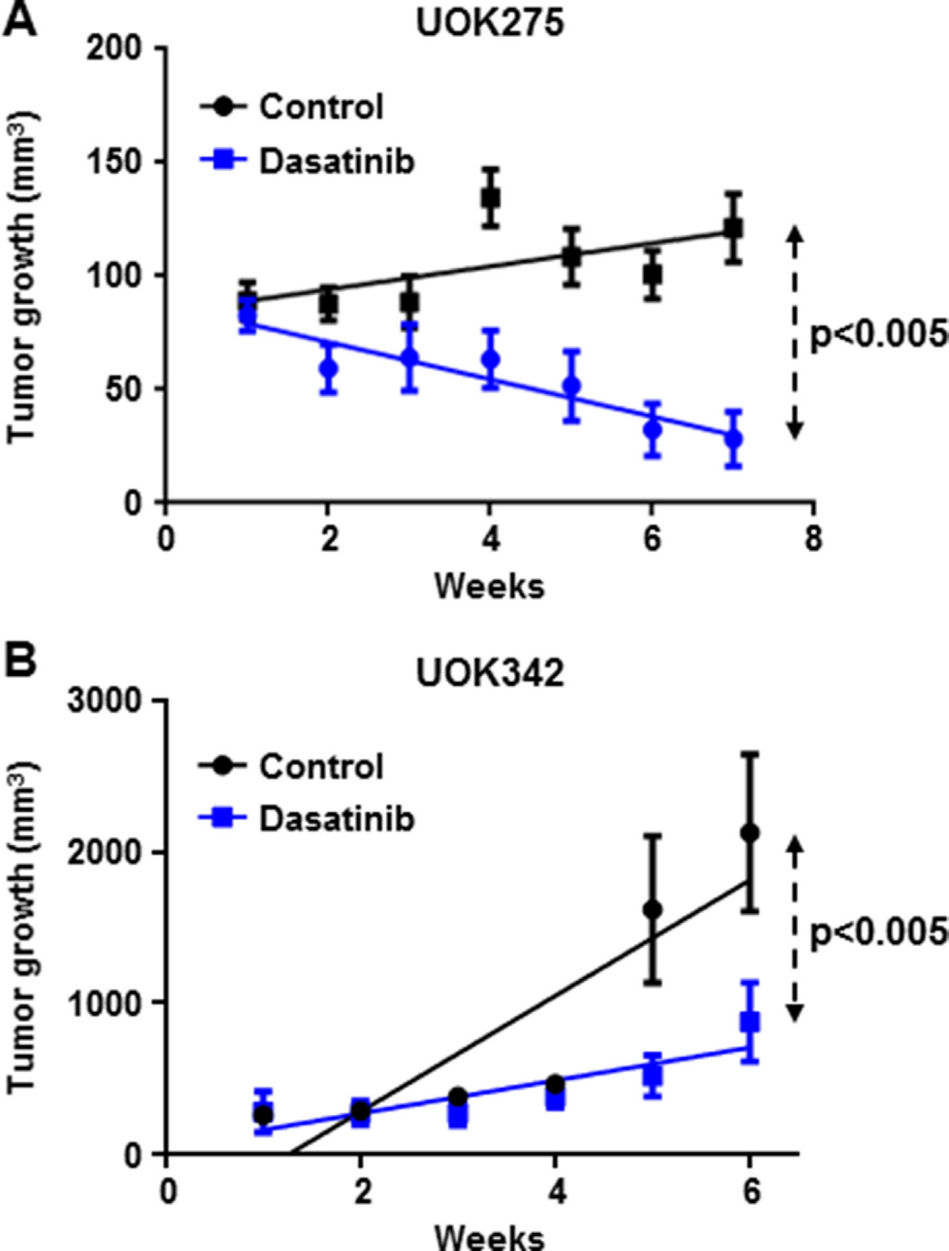
Dasatinib inhibits *NF2*-deficient tumor growth in two xenograft animal models (**A**) Twenty female athymic nude mice were injected subcutaneously with 2 million of UOK275 cells. When the tumor volumes reached > 80 mm^3^, mice were randomized into 2 groups. One group was treated with dasatinib (25 mg/kg week days by oral gavage) while the other group received the vehicle (water/PEG 1:1) in a similar manner. Tumor volumes were measured once a week using calipers. Tumor volume was estimated using the formula v = lxL^2^. (**B**) Twenty female athymic nude mice were injected with 2 million of UOK342 cells. When the tumor volumes reached >100 mm^3^, mice were randomized into 2 groups treated either with dasatinib (25 mg/kg week days by oral gavage) or the vehicle (water/PEG 1:1). Tumor volumes were measured using calipers once a week. Tumor volume was estimated using the formula v = lxL^2^.

Next, we evaluated the *in vivo* effect of dasatinib on two PRCC *NF2*-deficient tumor xenograft models (UOK275 and UOK342). Female nude mice bearing UOK275 xenografts or UOK342 xenografts were treated with dasatinib by oral gavage (25 mg/kg/week days) or vehicle (water/PEG 1:1; 10 mice per groups). Tumors were measured on a weekly basis and, after six to seven weeks, tumors were excised and the mice sacrificed. As shown in Figure 6A and 6B, dasatinib treatment significantly inhibited tumor growth in both UOK275 xenografts and UOK342 xenografts, suggesting that targeted inhibition of Yes in YAP-activated tumors with dasatinib may present a therapeutic potential for patients with *NF2*-deficient PRCC tumors that have lost regulation by the Hippo signaling pathway.

## DISCUSSION

Papillary renal cell carcinoma (PRCC) is a genetically and histologically heterogeneous disease. The Cancer Genome Atlas Research Network's recent comprehensive analysis of the molecular and genetic features of PRCC has greatly improved our understanding of the pathways involved in PRCC carcinogenesis and is opening novel targeted therapeutic opportunities. Mutation of *NF2*, a key regulator of the Hippo signaling pathway was observed in 3.6% of PRCC and, in combination with copy number loss, *NF2* alterations were present in 22.5% PRCC, including both type 1 and type 2 PRCC histologies [2].

We identified 2 PRCC cell lines bearing *NF2* mutations; one derived from a patient with type 2 PRCC, UOK275, and one derived from a patient with type 1 PRCC, UOK342. In addition, a known, commonly used type 1 PRCC cell line, ACHN, had been reported to have a *NF2* mutation and this was confirmed within this study. A series of siRNA experiments demonstrated that all 3 *NF2*-deficient PRCC cell lines were dependent on YAP1 transcriptional activity to support their survival and growth. Similar to that observed within β-catenin active tumors [14], Yes activation and expression were also critical for the survival of these *NF2*-deficient PRCC cell lines and for YAP1 transcriptional activity. YAP1 transcriptional activity is regulated at the posttranslational level by phosphorylation by multiple kinases, including both the downstream members of the Hippo signaling pathway and several members of the src kinase family, including Yes [25–27]. Dasatinib and saracatinib are src inhibitors that have a broad inhibitory effect on most of the src family members, including Yes. While alterative src inhibitors, such as WH-4-023 and PP2, effect similar members of the src kinase family, but are not reported to inhibit Yes. Comparative analysis using these two different sets of src inhibitors demonstrated a specific effect viability and expression of YAP1 downstream targets from the Yes kinase inhibitors and no effect from the src inhibitors that lacked Yes kinase inhibition. This strengthened our hypothesis that these *NF2*-deficient PRCC cell lines could be specifically targeted with dasatinib and saracatinib in a predominantly Yes-dependent manner. These data were further complemented by showing that Yes kinase inhibitors induced a G0/G1 arrest in all 3 *NF2*-deficient PRCC cell lines, but had no effect on the HEK293 control cell lines, while the alternative src inhibitors had no effect on cell cycle in any cell line.

The Hippo pathway controls organ size and cell proliferation by regulation of YAP1 cellular localization and transcriptional activity. In *NF2*-deficient PRCC, the disruption of the Hippo pathway and aberrant activation of YAP1 are likely to promote cell proliferation. The YAP1 downstream targets cyclin D1 (*CCDN1*) and survivin (*BIRC5*) could both regulate the cell cycle. Cyclin D1 expression is required and maintained during the G1 phase and for the initiation of S phase, after which it is degraded. Survivin is part of the inhibitor of apoptosis (IAP) family and is only expressed in G2/M phase. It is thought that survivin affects the M phase by binding to mitotic spindles. The G0/G1 arrest observed in the treated cell lines is most likely due, at least in part, to the decrease in cyclin D1 expression.

The *in vitro* analysis had demonstrated the effectiveness of Yes inhibitors on cell lines, so we proceeded to assess the effect of dasatinib *in vivo* on the growth of two *NF2*-deficient PRCC xenograft models using the type 2 PRCC cell line UOK275 and the type 1 PRCC cell line UOK342. Both models presented a tumor growth inhibition in the dasatinib-treated groups compared with the vehicle-treated groups. Taken together our data identify both Yes kinase activity and YAP1 transcriptional activity as attractive therapeutic targets in *NF2*-deficient PRCC.

The recent uncovering of a subgroup of PRCC patients with *NF2* mutation has paved the way for the development of novel targeted therapies. This preclinical study is providing some scientific rationale as to why to target Yes kinase activity and identifies Yes inhibitors (such as dasatinib) as promising potential therapeutic agents for patients with *NF2*-deficient PRCC tumors.

## MATERIALS AND METHODS

### Cell lines and cell culture

UOK275 and UOK342 cell lines were established in the Urologic Oncology Branch from tumor specimens (National Cancer Institute, Bethesda, MD). A full characterization of the cell lines will be published elsewhere. Briefly, UOK275 was established from a 10 cm retroperitoneal mass that proved to be metastatic RCC in a 55-year-old male who had undergone a right radical nephrectomy the year before for multifocal renal masses involving a 9 cm area of the right kidney and invading the renal vein. UOK342 was established from ascites fluid taken from a 63-year-old male with a history of type 1 PRCC. The immortalized human embryonal kidney cell line HEK293 and the papillary kidney tumor cell line ACHN were both purchased from ATCC (Manassas, VA, USA). Cells were cultured in high glucose DMEM containing pyruvate and glutamine and supplemented with 10%FBS. The cells were harvested or treated when they reached 70–80% confluence.

### Cell line mutation identification and gene sequencing

DNA was extracted from cell pellets using a Promega Maxwell 16 Cell DNA Purification Kit (Promega, WI, USA). UOK275 and UOK342 cell line DNA was assessed using the OncoVar assay as previously described [28, 29]. Mutations in *NF2* were validated by PCR using a Qiagen Taq PCR Core Kit (Qiagen, MD, USA) per the manufacturer's specifications, followed by bidirectional sequencing using the BigDye Terminator v.1.1 Cycle Sequencing Kit (Applied Biosystems, CA, USA) per the manufacturer's specifications. Sequencing was performed on an ABI 3730/ABI 3130xl Genetic Analyzer automated sequencing machine (Applied Biosystems, CA, USA). Sanger Sequencing was conducted at the CCR Genomics Core at the National Cancer Institute. Forward and reverse sequences were evaluated using Sequencher 5.4.6 (Genecodes, MI, USA). *NF2*-specific primers used were exon 2F: GAGAGTGGAGAGTGCAGAGA, exon 2R: CTTCTCTACTATACAGCTACAG, exon 9F: GGTTTAGTGCCTGGATACTG, exon 9R: CTTCACAAGATGTCACTCTGA, exon 12F: ACAGCACATGATCCCACTTC, exon 12R: GACAACTGCTGTAGAGCTCA.

### Reagents

Dasatinib, sarcatinib, PP2 and WH-4-023 were purchased from Selleck Chemicals (Houston, TX, USA). Smartpool ON-TARGETplus siRNAs targeting YAP and YES were purchased from GE Healthcare (Pittsburgh, PA, USA).

### Immunoblotting

Ten to twenty micrograms of protein were loaded in 4–20% polyacrylamide gels (Biorad, Hercules, CA, USA). After electrophoresis, proteins were transferred to PVDF membranes, blocked with 5% fat-free milk for 1h, and incubated with primary antibodies overnight at 4°C (dilution 1:1000). After 3 × 10 min washes with TBS-Tween, blots were incubated with horseradish peroxidase-linked secondary antibodies (1:3000 dilution in 2% fat-free milk; Biorad) for 2 h before development with the ECL protein detection system (Thermo Fisher Scientific, Rockford, IL). Rabbit antibodies against survivin (#2808), YAP (#4912), and cyclin D1 (#2922), and mouse antibodies against β-actin (#3700) and α-tubulin (#3873) were from Cell Signaling Technology, Inc (Danvers, MA, USA). Rabbit antibody against CTGF (#ab5097) and phospho-YAP (Tyr357) (#ab62751) was from Abcam (Cambridge, MA, USA).

### Cell viability and siRNA transfections

Cell viability was measured using a Cell-Titer Glo purchased from Promega Biosciences, Inc. (San Luis Obispo, CA, USA) following the manufacturer's protocol and as previously described [30].

### RNA extraction and Real-Time PCR analysis

Cell lines were grown to approximately 80–90% confluency, washed with 5 mL of sterile PBS, and RNA was extracted using the RNeasy mini kit (Qiagen, MD) in accordance with the manufacturer's protocol. RNA was eluted in 50 μL of RNase-free water and the measured using a NanoDrop 2000 UV-Vis Spectrophotometer (Thermo Fisher Scientific Inc., MA, USA). Complementary DNA was generated from 2 μg of RNA using the SuperScript^®^ VILO™ cDNA Synthesis Kit (Invitrogen, CA, USA) in a 20 μL volume and diluted 10-fold with RNase-free water for downstream applications. RT-PCR amplification was performed using an ABI ViiA7 real-time PCR system (Thermo Fisher Scientific Inc., MA, USA) and TaqMan^®^ Gene Expression Assays (Thermo Fisher Scientific Inc., MA) using 2 μL of dilute cDNA for each reaction. All reactions were performed in triplicate using the standard ViiA7 reaction template and expression was normalized to the 18S control gene (Hs99999901_m1) and calculated using the ViiA7 software as comparative CT (ΔΔCT) values. The following set probes were used: *BIRC5* (Hs03043574_m1), *CTGF* (Hs01026927_g1), and *CCND1* (Hs00765553_m1).

### YAP nuclear/cytoplasmic localization analyses

Two thousand cells were seeded in 96-black well plates (Corning). The following day, cells were fixed with 4% paraformaldehyde for 1 hour and permeabilized with 1% triton for 10 minutes. After blocking with 1% bovine serum albumin (BSA)/PBS for 1 hour, the cells were incubated overnight with FITC-conjugated rabbit YAP antibody (Cell Signaling #14729) in 0.1% BSA/PBS at 4°C. After being washed with PBS, cells were incubated 10 mg/mL Hoechst 33342 and Texas Red phalloidin (Invitrogen) in 0.1% BSA/PBS for 1 hour. Cells were washed 3 times with PBS prior to image acquisition. Image acquisition for each well was performed on an IN Cell Analyzer 2000 (GE Healthcare) by using a 10x objective lens. YAP nuclear/cytoplasmic localization was determined with the IN Cell Developer software (GE Healthcare) by using Hoechst staining to define nuclear regions and phalloidin staining to define cells cytoplasm.

### Flow cytometry analysis of cell cycle

Cells were incubated with 10 μM BrdU for 2 hours prior harvest. Cells were then trypsinized and fixed overnight at 4°C in 70% ethanol in calcium- and magnesium-free phosphate buffered saline (PBS). Ethanol solution was removed and cells were incubated in 3 mL of 0.08% pepsin in 0.1 N HCl at 37°C for 20 minutes. Pepsin was removed and nuclei were incubated in 1.5 mL of 2 N HCl at 37°C for 20 minutes. The nuclei containing acid solution was neutralized with 3 mL of 0.1 M sodium borate. Nuclei were spun out of neutralized acid and washed with 2 mL IFA buffer (10 mM HEPES pH 7.4, 150 mM NaCl, 4% FBS and 0.1% sodium azide with 0.5% Tween-20 added on the day of use) and then incubated overnight at 4°C with anti-BrdU clone MoBU-1 conjugated to AlexaFluor488 (Invitrogen B35130) in IFA buffer. DNA was stained for 30 minutes with IFA buffer with of 50 μg/mL propidium iodide (PI) (Sigma-Aldrich #P4864) and 5 μg/mL RNase A (Sigma #R4642). Cell cycle analysis (bivariate plots of BrdU incorporation and DNA content) was performed on a FACSCanto II (Becton Dickinson). Data were collected and analyzed using FlowJo software (FlowJo, Ashland, OR, USA).

### Animal study

Animal experiments were performed in accordance with the guidelines of the Animal Care and Use Committee of the National Institutes of Health. Forty female athymic nude mice (Taconic, Germantown, NY, USA) were injected on the right flank with 2 million of UOK342 or UOK275 cells diluted in matrigel (100%; BD Bioscience, San Jose, CA, USA); 20 mice per cell lines). Two weeks after injection, tumor volume reached > 100 mm^3^. Mice were randomized into four groups of ten mice bearing either UOK342 or UOK275. One group of UOK342 and one group of UOK275 were treated with dasatinib (25 mg/kg week days by oral gavage) while the other groups received the vehicle (water/PEG 1:1) in a similar manner. After 6-7 weeks treatment, animals were sacrificed.

### Statistics

All values are expressed as mean ± standard error. All experiments have been performed three times, with exception of the animal study. Values were compared using the Student-Newman-Keul's test. *P* < 0.05 was considered significant.

## SUPPLEMENTARY MATERIALS FIGURES AND TABLES


