# Supplementary material for: Targeting loss of the Hippo signaling pathway in NF2-deficient papillary kidney cancers
Source: Oncotarget. 2018 Jan 10;9(12):10723–33. doi: 10.18632/oncotarget.24112 (PMC5828210; doi:10.18632/oncotarget.24112)
Supplement: Supplementary file 1 [file oncotarget-09-10723-s001.pdf]

Targeting loss of the Hippo signaling pathway in *NF2*-deficient papillary kidney cancers

SUPPLEMENTARY MATERIALS

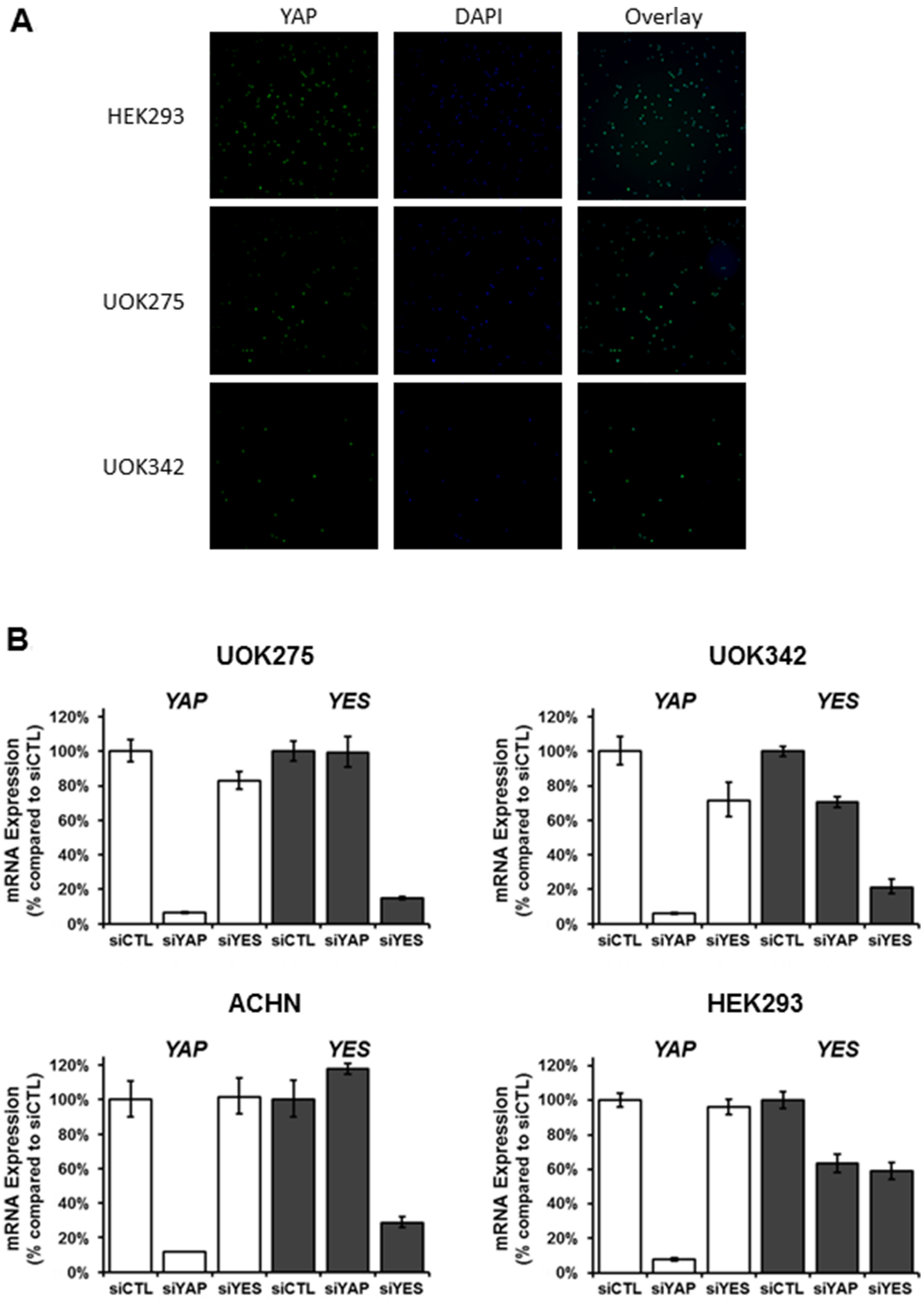

Supplementary Figure 1: (**A**) Representative images of YAP staining (green) and DAPI (blue) are shown for HEK293, UOK275 and UOK342. (**B**) The efficiency of transient silencing of *YES1* and *YAP1* by small interference RNA or mock siRNA was assessed in UOK275, UOK342, ACHN and HEK293 24 hours post-transfection by TaqMan assay. siCTL: mock siRNA; siYES: siRNA against *YES1*; siYAP: siRNA against *YAP1*.

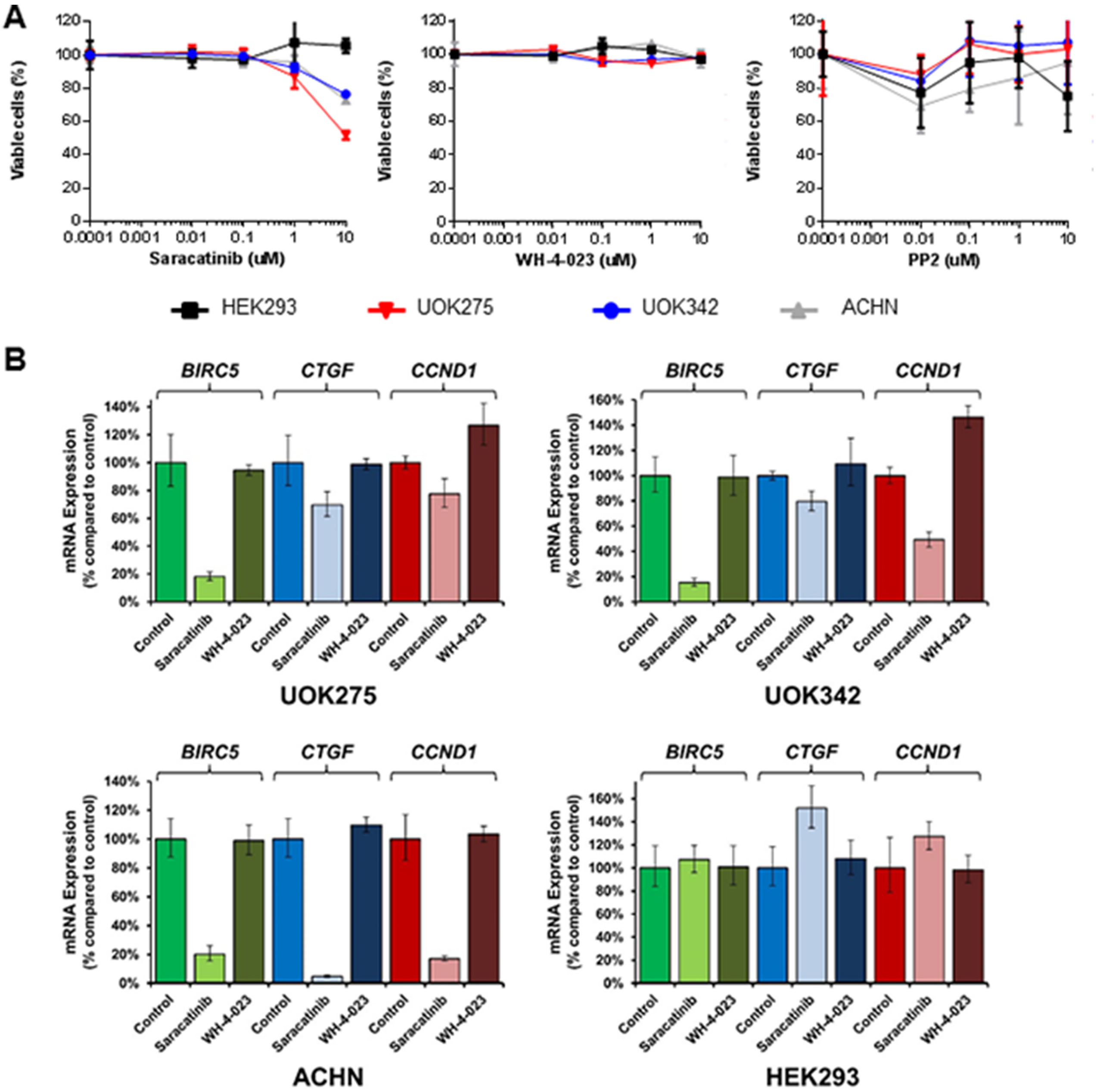

Supplementary Figure 2: (**A**) Dose concentration treatment of saracatinib, WH-4-023 and PP2 (μM) on the viability of UOK275, UOK342 and ACHN. HEK293 were used as controls. Cells were treated as indicated for 48 hours then viability was assessed by Cell-Titer Glo assay. (**B**) Expression of *BIRC5* (survivin), *CTGF* (CTGF) and *CCND1* (cyclin D1) was assessed by TaqMan assay 24 hours after saracatinib or WH-4-023 treatment (100nM) in HEK293, UOK275, UOK342 and ACHN.

Supplementary Table 1: Effect of dasatinib on cell cycle

	Dasatinib	G0/G1 (%)		S (%)		G2/M (%)	
		Avg	SE	Avg	SE	Avg	SE
HEK293	0 nM	39.4	2.0	48.4	1.6	8.4	0.4
	50 nM	45.2	4.2	40.8	6.2	9.5	1.1
	500 nM	46.8	2.4	41.7	3.0	7.1	0.6
UOK275	0 nM	67.8	4.5	21.0	2.8	9.8	2.1
	50 nM	83.2	1.4	3.7	0.4	11.6	1.3
	500 nM	84.8	2.4	1.4	0.6	12.3	2.1
UOK342	0 nM	73.4	4.2	16.2	5.4	7.9	0.5
	50 nM	86.0	0.9	4.2	2.6	6.5	1.3
	500 nM	89.6	3.7	2.3	0.9	6.1	2.3
ACHN	0 nM	45.6	4.7	32.8	2.5	16.4	2.0
	50 nM	58.9	6.7	21.5	4.9	13.8	1.1
	500 nM	67.5	5.8	6.0	3.0	16.4	2.5

Supplementary Table 2: Effect of saracatinib on cell cycle

	Saracatinib	G0/G1 (%)		S (%)		G2/M (%)		*N*
		Avg.	SE	Avg.	SE	Avg.	SE	
HEK293	0 nM	50.8	0.259808	41	0.173205	5.1	0.5	3
	100 nM	48.5	0.173205	42	1.529978	6.5	1.7	3
UOK275	0 nM	50	0	44	0	5.1	0	2
	100 nM	83.3	0.565685	9.1	0.233345	7	0.3	2
UOK342	0 nM	37.4	0	60.3	0	1.6	0	2
	100 nM	43.2	0.53033	55.1	0.777817	1.3	0	2
ACHN	0 nM	45.3	0	41.9	0	7.1	0	4
	100 nM	46.8	0.725	43.5	0.8	6	0.5	4

Supplementary Table 3: Effect of PP2 on cell cycle

	PP2	G0/G1 (%)		S (%)		G2/M (%)		*N*
		Avg.	SE	Avg.	SE	Avg.	SE	
HEK293	0 nM	50.9	0.244949	40.9	0.163299	4.9	0.4	3
	100 nM	50.1	0.830439	35.4	2.600997	9.6	0.6	3
UOK275	0 nM	50	0	44	0	5.1	0	2
	100 nM	56.4	2.05061	37.5	1.697056	5.5	0.2	2
UOK342	0 nM	37.4	0	60.3	0	1.6	0	2
	100 nM	41	1.237437	57.1	1.131371	1.2	0.1	2
ACHN	0 nM	48.8	1.752632	38.8	1.537602	9.2	1	4
	100 nM	51.7	3.793003	39.4	5.024005	7.8	1.6	4

Supplementary Table 4: Effect of WH-4-023 on cell cycle

	WH-4-023	G0/G1 (%)		S (%)		G2/M (%)		*N*
		Avg.	SE	Avg.	SE	Avg.	SE	
HEK293	0 nM	50.8	0.259808	41	0.173205	5.1	0.5	3
	100 nM	49.3	0.606218	37.3	2.569209	8.8	0.2	3
UOK275	0 nM	50	0	44	0	5.1	0	2
	100 nM	53.7	1.59099	40.6	1.52028	5	0.3	2
UOK342	0 nM	37.4	0	60.3	0	1.6	0	2
	100 nM	40.9	0.070711	56.9	0.070711	1.6	0	2
ACHN	0 nM	48.8	1.752632	38.8	1.537602	9.2	1	4
	100 nM	52.8	1.897819	37.4	3.014444	8.4	1.8	4
